# Comparison of different methods for the estimation of aortic pulse wave velocity from 4D flow cardiovascular magnetic resonance

**DOI:** 10.1186/s12968-019-0584-x

**Published:** 2019-12-12

**Authors:** Sophia Houriez--Gombaud-Saintonge, Elie Mousseaux, Ioannis Bargiotas, Alain De Cesare, Thomas Dietenbeck, Kevin Bouaou, Alban Redheuil, Gilles Soulat, Alain Giron, Umit Gencer, Damian Craiem, Emmanuel Messas, Emilie Bollache, Yasmina Chenoune, Nadjia Kachenoura

**Affiliations:** 1 Sorbonne Université, INSERM, CNRS, Laboratoire d’Imagerie Biomédicale (LIB), 75006 Paris, France; 2 ESME Sudria Research Lab, Paris, France; 3 Institute of Cardiometabolism and Nutrition (ICAN), Paris, France; 4 Hopital Européen Georges Pompidou, Paris, France; 5 CMLA, ENS Cachan, CNRS, Université Paris-Saclay, 94235 Cachan, France; 6 Universidad Favaloro-CONICET, IMeTTyB, Buenos Aires, Argentina

**Keywords:** Pulse wave velocity, 4D flow CMR, Aortic stiffness, Aging

## Abstract

**Background:**

Arterial pulse wave velocity (PWV) is associated with increased mortality in aging and disease. Several studies have shown the accuracy of applanation tonometry carotid-femoral PWV (Cf-PWV) and the relevance of evaluating central aorta stiffness using 2D cardiovascular magnetic resonance (CMR) to estimate PWV, and aortic distensibility-derived PWV through the theoretical Bramwell-Hill model (BH-PWV). Our aim was to compare various methods of aortic PWV (aoPWV) estimation from 4D flow CMR, in terms of associations with age, Cf-PWV, BH-PWV and left ventricular (LV) mass-to-volume ratio while evaluating inter-observer reproducibility and robustness to temporal resolution.

**Methods:**

We studied 47 healthy subjects (49.5 ± 18 years) who underwent Cf-PWV and CMR including aortic 4D flow CMR as well as 2D cine SSFP for BH-PWV and LV mass-to-volume ratio estimation. The aorta was semi-automatically segmented from 4D flow data, and mean velocity waveforms were estimated in 25 planes perpendicular to the aortic centerline. 4D flow CMR aoPWV was calculated: using velocity curves at two locations, namely ascending aorta (AAo) and distal descending aorta (DAo) aorta (S1, 2D-like strategy), or using all velocity curves along the entire aortic centreline (3D-like strategies) with iterative transit time (TT) estimates (S2) or a plane fitting of velocity curves systolic upslope (S3). For S1 and S2, TT was calculated using three approaches: cross-correlation (TTc), wavelets (TTw) and Fourier transforms (TTf). Intra-class correlation coefficients (ICC) and Bland-Altman biases (BA) were used to evaluate inter-observer reproducibility and effect of lower temporal resolution.

**Results:**

4D flow CMR aoPWV estimates were significantly (*p* < 0.05) correlated to the CMR-independent Cf-PWV, BH-PWV, age and LV mass-to-volume ratio, with the strongest correlations for the 3D-like strategy using wavelets TT (S2-TTw) (*R* = 0.62, 0.65, 0.77 and 0.52, respectively, all *p* < 0.001). S2-TTw was also highly reproducible (ICC = 0.99, BA = 0.09 m/s) and robust to lower temporal resolution (ICC = 0.97, BA = 0.15 m/s).

**Conclusions:**

Reproducible 4D flow CMR aoPWV estimates can be obtained using full 3D aortic coverage. Such 4D flow CMR stiffness measures were significantly associated with Cf-PWV, BH-PWV, age and LV mass-to-volume ratio, with a slight superiority of the 3D strategy using wavelets transit time (S2-TTw).

## Background

Aortic stiffening is an early sign of remodeling and functional changes in arterial hemodynamics, and a marker of cardiovascular aging [1, 2]. The clinical usefulness of aortic stiffness has been previously demonstrated through its significant associations with adverse left ventricular (LV) remodeling, coronary heart disease, atherosclerosis and elevated mortality [3–6]. Aortic stiffness is commonly assessed using carotid-femoral (Cf) pulse wave velocity (PWV), which has been shown to be an accurate non-invasive alternative [7–9] to cardiac catheterization [10] in the in vivo measurement of pulse wave velocity (PWV). PWV is defined as the distance (D) travelled by the pressure wave between two anatomical locations, divided by the transit time (TT) spent by the wave to travel such distance.

Cardiovascular magnetic resonance imaging (CMR) offers excellent anatomical coverage and its anatomical and velocity-encoded sequences allow an accurate estimation of aortic geometry (length, diameters, volumes) as well as blood flow-derived indices in the thoracic aorta. In particular, two-dimensional through-plane phase-contrast CMR (2D phase contrast (PC)-CMR) has been used for the estimation of aortic (ao) PWV (aoPWV), using arch length from the ascending (AAo) to the descending (DAo) aorta, divided by the TT derived from a single acquisition plane positioned perpendicularly to both the AAo and DAo [11–14]. Alternatively, ascending aorta PWV estimation was also proposed using the theoretical Bramwell-Hill (BH) model and aortic distensibility, which is commonly derived from aortic cine CMR and central pulse pressure [15–17].

CMR with full three-dimensional anatomical coverage and velocity encoding in the three directions resolved throughout the cardiac cycle (4D flow CMR) has been developed, opening new and unique opportunities to both visualize and quantify cardiovascular complex blood flow [18, 19]. 4D flow CMR has several advantages, including its excellent 3D anatomical and velocity coverage which enables an accurate estimation of aortic arch length and aortic flow rates and velocities. Furthermore, as compared with 2D PC-based approaches, 4D flow PWV is better suited to diseases with complex arterial geometry and tortuosity or with heterogeneous stiffness patterns along the arterial tree that can be associated with atherosclerosis [20] or changes in arterial size [21, 22]. Moreover, the estimation of aortic PWV from 4D flow CMR [20] has been shown to be feasible using either TT [20, 23–25] or plane fitting [20] approaches. The present study aims to provide a comprehensive comparison of both TT and plane fitting-based methods for aortic PWV estimation from 4D flow CMR in healthy subjects, in terms of: 1) associations with the CMR-independent well-established Cf-PWV measure, 4D flow CMR-independent BH-PWV, age and LV mass-to-volume ratio, as well as 2) inter-observer reproducibility and robustness to temporal resolution.

## Methods

### Study population and data acquisition

We retrospectively studied 47 healthy subjects 20 to 79 years (49.5 ± 18 years, 23 males), without overt cardiovascular disease. Approval of the local Institutional Review Board and informed consent was obtained from all participants. All subjects had a CMR exam on a 3 T system (Discovery 750w GEM, General Electric Healthcare, Waukesha, Wisconsin, USA), including 4D flow acquisitions with retrospective electrocardiogram (ECG) gating in a sagittal oblique volume encompassing the thoracic aorta. The three-directional encoding velocity was equal to 250 cm/s and the scan parameters were as follows: flip angle = 15°, reconstructed voxel size = 1.5 × 2.4 × 1 mm^3^, echo time = 1.7 ms, repetition time = 4.3 to 4.4 ms, acquisition matrix = 256 × 96 × 136 and views per segment = 2, resulting in acquired temporal resolution = 34.4 to 35.2 ms, which was then reconstructed into 50 frames per cardiac cycle while applying a view sharing technique. Acquisition time was around 10 min. Under-sampled k-space data were reconstructed using an algorithm combining iterative autocalibrating parallel imaging and compressed sensing with nonlinear ℓ1-norm wavelet regularization (L1 SPIR-iT) [26–28] with a factor of acceleration of 2 for the slice direction and 2.4 for the phase direction. 4D flow CMR datasets of 13 randomly selected subjects (44 ± 13 years) were also reconstructed using 20 frames per cardiac cycle to study the effect of temporal resolution on aortic PWV estimation. Injection of 0.20 mmol/kg gadolinium contrast agent (gadobenate dimeglumine, Guerbet, France) was performed just prior to 4D flow CMR acquisition.

Blood pressures were measured using a Sphygmocor Xcel (AtCor Medical, Australia) device simultaneously to CMR acquisitions, and central systolic (SBP) and diastolic (DBP) blood pressures were recorded. Pulse pressure (PP=SBP-DBP) was subsequently calculated. All subjects also underwent an applanation tonometry exam immediately after CMR acquisitions to compute the Cf-PWV, as previously described [29], to provide an CMR-independent arterial PWV measure.

In addition, cine balanced steady-state free precession (bSSFP) images were acquired during breath-holds perpendicular to the aorta at the level of pulmonary artery bifurcation to simultaneously image both AAo and DAo as well as in short axis views to cover the whole LV, using the following scan parameters: acquisition matrix = 260 × 192, repetition time = 3.7 ms, echo time = 1.5 ms, flip angle = 50°, pixel size = 0.74 mm × 0.74 mm, slice thickness = 8 mm, views per segment = 12. LV data were analysed using Qmass 6 Software (Medis, Leiden, the Netherlands) while semi-automatically tracing endocardial and epicardial contours on all contiguous short axis slices, resulting in LV end-diastolic (EDV) and end-systolic (ESV) volumes as well as LV mass (LVM). The LV mass-to-volume ratio (LVM/EDV) was calculated and used as a measure of LV remodeling [30].

Aortic bSSFP images were analysed using the validated ArtFun software (Sorbonne Université/U.1146 Inserm) [31], which automatically delineated AAo lumen contours for all phases of the cardiac cycle. Then, local AAo BH-PWV was calculated from AAo distensibility (AAo systolic area-AAo diastolic area)/(AAo diastolic area · central PP) according to the Bramwell-Hill (BH) model [15–17] i.e. BH-PWV = 1/ $\sqrt{\uprho \times \mathrm{distensibility}}$ where ρ is blood density set to 1060 kg.m^− 3^.

### Estimation of aortic pulse wave velocity from 4D flow CMR data

**Fig. 1:**
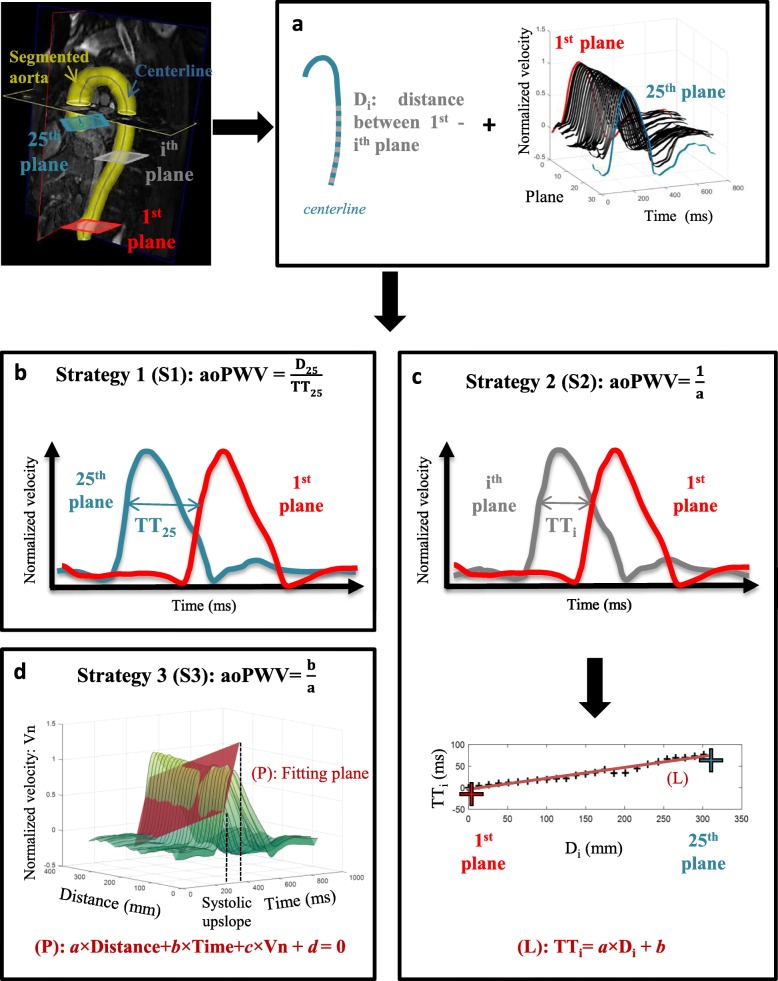
Aortic pulsed wave velocity (PWV) estimation from 4D flow CMR. **a** Shared pre-processing step for all aortic PWV (aoPWV) strategies: aortic 3D segmentation, positioning of cross-sectional planes perpendicular to the aortic centreline between the distal descending aorta (1st plane) and proximal ascending aorta (25th plane), extraction of the corresponding 25 mean velocity curves, as well as Di distances between the 1st and the ith planes along the centreline. Of note, mean velocity curves were interpolated with a 1-ms time step using a spline function. **b** For strategy 1 (S1), aoPWV was defined as the ratio of the distance (D_25_) between the most distal (#1) and most proximal (#25) planes to the transit time (TT_25_) estimated from the corresponding normalized velocity waveforms. Transit time estimation methods are described in Fig. 2. **c** For strategy 2 (S2), intermediate distances (Di) and transit times (TT_i_) were estimated as in S1 between plane 1 and each successive plane (#i), until the proximal ascending aortic plane is reached. Such process provides the plot at the bottom, which was then linearly fitted (L: TT_i_ = a*D_i_ + b). aoPWV was equal to reciprocal of the slope (**a**). **d** For strategy 3 (S3), transit time estimation was not needed since aoPWV was defined from the parameters of the plane (P: a × distance+b × time + c × velocity + d = 0) used to fit the systolic upslope of the normalized velocity curves corresponding to the 25 aortic planes illustrated in panel **a**

Background phase offsets and phase wrapping were corrected as previously recommended [23]. Besides, to improve the quality of aortic segmentation, a 3D PC CMR angiogram was computed [32] while combining the modulus and velocity images averaged over time phases around the systolic peak, defined as the temporal phase with maximal velocity in the AAo. Aortic volume segmentation from the PC-CMR angiogram was then used to mask velocities through the cardiac cycle and to extract the aortic centerline and subsequently aortic length, using a semi-automated custom segmentation software (Mimosa, Sorbonne Université/Inserm 1146) [33]. Finally, 25 planes which were equally spaced and automatically positioned perpendicularly to the aortic centerline between the distal DAo and AAo, were used to calculate 25 mean velocity curves along the aorta which were interpolated with a time step of 1 ms using a spline function (Fig. 1a).

Three different strategies were then used for aoPWV estimation. While the first strategy (Strategy 1) was equivalent to a 2D PC-CMR strategy based on two anatomical locations, strategy 2 and 3 included all the 3D information.

#### Strategy 1

aoPWV was computed as the distance (D) along the centerline between two extreme planes located in AAo and distal DAo, divided by TT calculated from normalized mean velocity curves at these two locations: aoPWV = D/TT (Fig. 1b). Of note, methods used for TT estimation are described below. Contrary to a through-plane 2D PC-CMR PWV measurement, a more extended segment of the aorta was included to keep PWV Cramer-Rao lower error bound (σ_*c*_) reasonable, and thus to minimize the error on PWV estimation [12] while considering image characteristics such as temporal and spatial resolutions as well as signal to noise ratio.

$$ {\upsigma}_c\ge \frac{c2}{SNR\times {\varphi}_{max}}\sqrt{\frac{6\times \Delta  t\times \Delta  x\times {t}_{rise}}{L^3}} $$

σ_c_:Cramer-Rao lower error bound; c: estimation of PWV; ∆t, ∆x : temporal, spatial resolution, respectively; t_rise_: systolic upslope duration; SNR: signal to noise ratio; φ_max_: phase angle at the maximal velocity; L: vessel length.

#### Strategy 2

aoPWV estimation used the full 3D anatomical coverage [24, 34]. First, intermediate distances (D_i_) along the aortic centreline were obtained while considering the distal DAo plane and each successive plane until proximal AAo was reached (Fig. 1a). Second, the corresponding paired normalized mean velocity waveforms were used for TT_i_ estimation. Such intermediate distances and transit times were plotted as TT_i_ according to D_i_ and, aoPWV was equal to the slope of the linear regression plot (Fig. 1c). Of note, methods used for TT_i_ estimation are described below.

#### Strategy 3

aoPWV was estimated using the full 3D anatomical coverage of the aorta as well, but required no TT calculation [20]. First, normalized mean velocity curves in aortic planes positioned between the proximal AAo and distal DAo were plotted according to time and distance along the aortic centreline (Fig. 1a and d). Then, aoPWV was calculated from the parameters of the plane used to fit the upslope of these curves, which was restricted between the 2nd and 8th decile of the systolic upslope.

### Transit time estimation

**Fig. 2:**
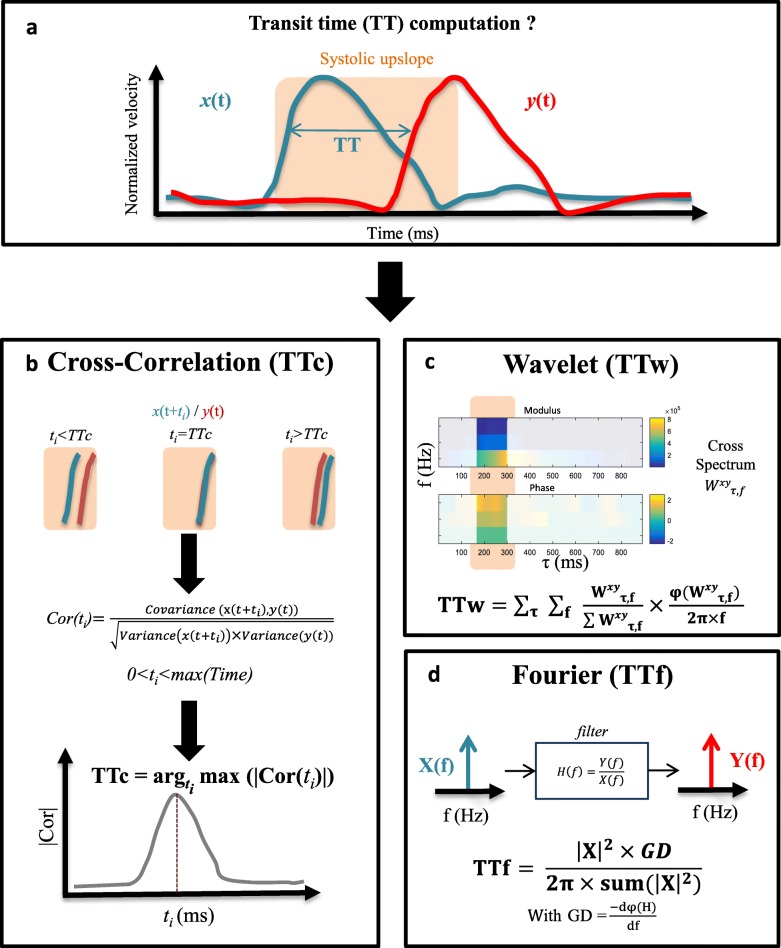
Transit time estimation from 4D flow CMR normalized time-resolved velocity curves. **a** Transit time (TT) is defined as the time shift needed to maximise the overlap between two normalized aortic velocity curves *x(t)* and *y(t)* extracted from two distinct planes positioned along the aortic centreline as illustrated in Fig. 1. Three TT estimation methods are used. **b** Cross-correlation transit-time (TTc): a time shift (t_i_), comprised between 0 and the systolic duration is applied to *x(t)* while maximizing its overlap with *y(t)* in terms of systolic upslope. Such overlap was iteratively defined by the cross-correlation Cor(t_i_) between the shifted curve *x(t + t*_*i*_*)* and *y(t)* systolic upslopes. TTc was set to the time shift maximizing the cross-correlation function Cor(t_i_). **c** Wavelet transform transit-time (TTw): 4th order Gaussian wavelet transform was applied on the normalized mean velocity curves *x(t)* and *y(t)* resulting in the provided modulus and phase of the cross-spectrum. TTw is then defined as the sum of such phase weighted by its modulus (see equation), while considering only the systolic upslope. **d** Fourier transform transit-time (TTf): time shift is modelled by the group delay introduced by a filter, which considers *x(t)* as an input and *y(t)* as an output. Normalized mean velocity curves *x(t)* and *y(t)* are Fourier transformed into (X(f) and Y(f)) defining the filter transfer function H(f) = Y(f)/X(f). TTf is calculated as the sum of the filter group delay (GD) weighted by the harmonics of the input signal (see equation)

For Strategy 1 and Strategy 2, three methods of TT estimation previously described in the literature [15, 25] were tested (Fig. 2). The first method (TTc) is based on cross-correlation which was shown to be reproducible, robust and reliable using 2D PC-CMR [35, 36]. The two other methods are based on wavelet (TTw) and Fourier (TTf) transforms, respectively, and were shown to be robust to poor temporal resolution [37, 38]. For all methods, TT is the time delay between two mean velocity curves or their systolic upslope measured at two different locations. Systolic upslope was restricted between its 2nd and the 8th decile. Technical details regarding TT estimates, which are described below, are provided in Fig. 2.

#### Cross-correlation transit time (TTc)

TT was equal, in the time domain, to the time shift which maximizes the correlation between the two mean velocity curves [39]. The measurement of TTc (Fig. 2b) was performed on normalized mean velocity curves while considering only the systolic upslope [40, 41] to minimize the effects of backward flow, which occurs mostly in late systolic or diastolic phases.

#### Wavelet transit time (TTw)

TT estimation in the time-frequency domain was derived from the 4-order complex Gaussian wavelet transform, which was initially applied to the normalized mean velocity curves at two aortic locations [37]. The complex cross-spectrum phase difference and modulus derived from such wavelet transforms were then restricted to the systolic upslope, similarly to the cross-correlation method. Finally, TTw was equal to the sum of the cross-spectrum phase difference weighted by its modulus (Fig. 2c) [37].

#### Fourier transit time (TTf)

TT estimation was modelled by the group delay of a filter which takes as input and output Fourier transforms (X(f) and Y(f)) of two normalized mean velocity curves (*x(t*) and *y(t)*, respectively) measured at two aortic locations. Such filter transfer function was calculated as H(f) = Y(f)/X(f). TTf was then equal to the group delay weighted by the harmonics of the input signal (Fig. 2d). In this frequency domain-based method [38], the entire time interval rather than systolic upslope was considered.

### Inter-observer reproducibility analysis

The only source of variability was the aortic segmentation, while the remaining steps for 4D flow CMR aoPWV estimation were fully automated. Accordingly, such segmentation along with computation of aoPWV using the above-mentioned strategies were performed on a subgroup of 15 randomly selected subjects (47 ± 22 years), in a random order by two independent operators (SHGS and KB with 2-year experience in 4D flow image processing), both blinded to subjects characteristics and to each other’s segmentation and quantitative results.

### Statistical analysis

Basic characteristics and PWV estimates are provided as median and interquartile range. The study population was divided into two subgroups according to age (< or ≥ 50 years) and Wilcoxon rank-sum test was used for comparison between the two age subgroups. Univariate linear regression was performed to study the relationships of 4D flow CMR aoPWV estimates, with Cf-PWV, BH-PWV, age, and LV mass-to-volume ratio. Regarding associations with LV mass-to-volume ratio, further multivariate models including potential confounders such as age, body mass index (BMI), SBP and gender were studied. Correlation coefficients R and estimated coefficient β are provided. Bland-Altman analyses were performed for: 1) comparison between 4D flow CMR methods and Cf-PWV as well as BH-PWV, to provide the positioning of 4D flow CMR measures against well-established PWV measures, 2) studying 4D flow CMR aoPWV inter-operator reproducibility and the effect of lower temporal resolution by comparing results of the 20 and 50 phases reconstructions, 3) comparison of the 4D flow CMR aoPWV method that was shown to be superior to the other methods based on the analyses described above, against the remaining 4D flow CMR aoPWV methods. For all Bland-Altman analyses, mean biases (MB), limits of agreements (LA) defined as mean bias±1.96 x Standard deviation [42] were provided. Intraclass correlation coefficients (ICC) were also calculated. Finally, the Wilcoxon rank-sum test was used to test for statistical significance of the differences between aoPWV values obtained by the two observers or for the two reconstructions. A result was reported as significant if *p* < 0.05. Statistical analyses were performed in Matlab (Mathworks, Natick, Massachusetts, USA).

## Results

Table 1 summarizes subjects characteristics. As expected, SBP and PP were significantly higher for older subjects when compared to younger subjects. The effect of age was also observed on Cf-PWV, BH-PWV and LV mass-to-volume ratio (LVM/EDV), which were all significantly higher in the older subjects. All 4D flow CMR aoPWV values are also presented in Table 1. They all showed a significant increase (*p* < 0.001) between the younger and older groups.

**Table 1:** Subjects characteristics and pulse wave velocity estimates

	20–49 year-old subjects (*N* = 25, *F* = 12)		50–79 year-old subjects (*N* = 22, *F* = 12)		All subjects (*N* = 47, *F* = 24)	
	Median	Interquartile range	Median	Interquartile range	Median	Interquartile range
Age (years)	35.0	27.7–43.7	67.5***	57.8–72.2	47.8	34.1–66.9
BMI (kg/m^2^)	22.2	20.6–24.8	24.6**	23.1–26.3	23.7	21.4–25.7
HR (bpm)	66.0	62–77	62.5	59.3–67.3	64.0	60–71
SBP (mmHg)	105	99–116	117**	113–122	114	103–119
DBP (mmHg)	77	70-83	81	74–86	78.7	73-85
PP (mmHg)	28	26-32	39***	32–43	32	28-40
LVM/ESV(g/ml)	1.1	1–1.3	1.3*	1–1.6	1.2	1–1.4
LVM/EDV(g/ml)	0.65	0.59–0.75	0.81*	0.64–0.92	0.72	0.62–0.87
Cf-PWV(m/s)	8.1	7.1–9	10.3***	9.6–11	9.5	7.9–10.3
BH-PWV(m/s)	4.9	4.0–5.2	9.4***	7.6–11.5	6.1	4.8–9.41
4D flow CMR aoPWV						
Strategy 1 - TTc (m/s)	5.8	5.1–7.5	9.3***	7.8–10.6	7.5	5.5–9.9
Strategy 1 - TTw (m/s)	6.4	5.5–8.2	11.1***	8.8–13.8	8.5	6.3–11.8
Strategy 1 - TTf (m/s)	5.5	4.7–6.4	7.8***	6.5–12.8	6.4	5.4–8.5
Strategy 2 - TTc (m/s)	4.9	4.7–6.5	8.0***	6.8–10	6.5	4.9–8.2
Strategy 2- TTw (m/s)	6.0	5.3–8.1	10.4***	8.6–12.5	8.4	5.9–10.4
Strategy 2- TTf (m/s)	5.7	5.2–6.4	8.2***	7.2–9.9	6.4	5.6–8.6
Strategy 3 (m/s)	5.5	5.1–7.7	9.2***	7.8–10.8	7.6	5.4–9.3

*N* number of subjects, *F* number of women, *BMI* body mass index, *HR* heart rate, *SBP* systolic blood pressure, *DBP* diastolic blood pressure, *PP* pulse pressure, *Cf-PWV* carotid-femoral pulse wave velocity, *LVM/ESV and LVM/EDV* left ventricular mass to end-systolic and end-diastolic volume ratios, *BH-PWV* ascending aorta pulse wave velocity according to the Bramwell Hill Model, *TTc, TTw and TTf* transit times estimated using cross-correlation, wavelets and Fourier transforms, respectively. Levels of significance were indicated by *** for *p* < 0.001, ** for *p* < 0.01, * for *p* < 0.05, for comparisons between the 2 age groups

### Association between 4D flow CMR aoPWV and Cf-PWV as well as BH-PWV

**Fig. 3:**
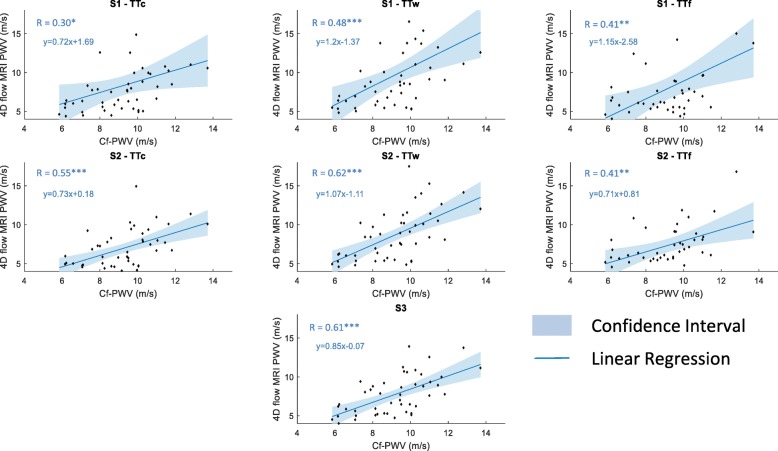
Associations between 4D flow CMR aortic PWV and carotid-femoral (Cf) PWV. Linear regressions (solid lines) and confidence intervals (shaded blue area) are displayed. Linear regression equations are provided. S1, S2 and S3: Strategies 1, 2 and 3. TTc, TTw and TTf: transit times estimated using cross-correlation, wavelets and Fourier transforms, respectively. Correlation coefficients (R) are provided along with their levels of significance, which were indicated by *** for *p* < 0.001, ** for *p* < 0.01, * for *p* < 0.05

Correlation coefficients for comparisons of all 4D flow-derived aoPWV estimates against reference tonometric Cf-PWV and ascending aorta BH-PWV are summarized in Table 2 and the corresponding linear regression plots are provided in Fig. 3. All 4D flow aoPWV estimates were significantly correlated (*p* < 0.05) with Cf-PWV as well as AAo BH-PWV, with the highest correlation coefficients obtained when using Strategy 2 along with the wavelet-based TT estimation (S2-TTw PWV: *R* = 0.62, *p* < 0.001 with Cf-PWV; *R* = 0.65, *p* < 0.001 with BH-PWV) and Strategy 3 (S3 PWV: *R* = 0.61, *p* < 0.001 with Cf-PWV; *R* = 0.64, *p* < 0.001 with BH-PWV).

**Table 2:** Associations of 4D flow CMR aoPWV methods with Cf-PWV, BH-PWV, age and LV mass-to-volume ratio

		Cf-PWV	BH-PWV	Age	LV mass-to-volume ratio
S1	TTc	0.30^*^	0.35^*^	0.44^**^	0.39^**^
	TTw	0.48^***^	0.56^***^	0.62^***^	0.44^**^
	TTf	0.41^**^	0.42^***^	0.39^**^	0.14
S2	TTc	0.55^***^	0.57^***^	0.66^***^	0.44^**^
	TTw	0.62^***^	0.65^***^	0.77^***^	0.52^***^
	TTf	0.41^**^	0.44^***^	0.53^***^	0.18
S3		0.61^***^	0.64^***^	0.76^***^	0.47^***^

*Cf-PWV* carotid-femoral (Cf) pulse wave velocity, *BH-PWV* ascending aorta pulse wave velocity according to the Bramwell Hill Model, *TTc, TTw and TTf* transit times estimated using cross-correlation, wavelets and Fourier transforms, respectively. Correlation coefficients are provided along with their levels of significance, which were indicated by *** for *p* < 0.001, ** for *p* < 0.01, * for *p* < 0.05

### Associations of 4D flow CMR aoPWV with age and left ventricular mass-to-volume ratio

Correlation coefficients for associations of all 4D flow CMR aoPWV values with age and with LV mass-to-volume ratio are shown in Table 2. All 4D flow-derived aoPWV measures were significantly associated with age, with the highest correlations obtained for S2-TTw (*R* = 0.77, *p* < 0.001) and S3 (*R* = 0.76, *p* < 0.001). Of note, such correlations were in the same range as the correlations between age and reference Cf-PWV (*R* = 0.72, *p* < 0.001) as well as BH-PWV (*R* = 0.79, *p* < 0.001). Interestingly, association with age remained significant even in the elderly group for both S2-TTw (*R* = 0.59, *p* < 0.001) and S3 (*R* = 0.59, *p* < 0.001) aoPWV measures. Significant correlations with age in univariate analysis remained significant (*p* < 0.001) after adjustment for BMI, gender and SBP without other significant correlates.

For associations with LV mass-to-volume ratio, 4D flow CMR aoPWV obtained using S2-TTw (*R* = 0.52, *p* < 0.001) and S3 (*R* = 0.47, *p* < 0.001) resulted in the highest correlations in univariate analysis. Such correlations were even slightly higher than those obtained for the reference Cf-PWV (*R* = 0.39, *p* < 0.01) and equivalent to those obtained for BH-PWV (*R* = 0.52, *p* < 0.001). The associations with LV mass-to-volume ratio obtained for S2-TTw, S3, and Cf-PWV remained significant after adjustment for age, gender, BMI, SBP (Table 3). Of note, gender was also a significant correlate.

**Table 3:** Independent correlates of left ventricular mass-to-volume ratio

	Model A		Model B		
	*β*	*R*	*β*	*R*	*Significant correlates*
Cf-PWV	0.038	0.39**	0.037	0.667***	Cf-PWV^*^, Gender^***^
S2-TTw	0.028	0.52***	0.024	0.672***	S2-TTw^*^, Gender^**^
S3	0.032	0.47***	0.027	0.670***	S3^*^, Gender^**^

*Cf-PWV* carotid-femoral pulse wave velocity, *S2-TTw* PWV estimated with Strategy 2 considering the, wavelets transform transit time, *S3* pulse wave velocity estimated with plane fitting. *β* estimated coefficient, *R* correlation coefficient. *Model A* univariate regression of association between pulse wave velocity measures with LV mass-to-volume ratio (LVM/EDV). *Model B* Model A + adjustment for age, gender (male), body mass index and systolic blood pressure. Levels of significance were indicated by *** for *p* < 0.001, ** for *p* < 0.01, * for *p* < 0.05

### Inter-observer reproducibility and effect of temporal resolution

Results of inter-observer reproducibility are summarized in the left part of Table 4. For all methods, there were no significant differences between 4D flow CMR aoPWV values measured by the two operators (*p* ≥ 0.38). Bland-Altman mean biases and limits of agreement indicated a higher reproducibility of the 3D-based strategies (S2 and S3), as compared with the 2D-based strategy (S1).

**Table 4:** Inter-observer reproducibility and effect of 20- vs. 50-time reconstructed frames temporal resolution

		Inter-observer reproducibility				Effect of temporal resolution			
		Mean bias (m/s)	LA (m/s)	p	ICC	Mean bias (m/s)	LA (m/s)	*p*	ICC
S1	TTc	−0.50	[−2.6;1.6]	0.59	0.95	−1.62	[−11;8.1]	0.49	0.47
	TTw	0.46	[−5.2;6.1]	1.00	0.86	0.29	[−1;1.5]	0.50	0.91
	TTf	−0.46	[−3.4;2.4]	0.43	0.91	0.35	[−0.5;1.2]	0.50	0.93
S2	TTc	0.06	[−0.6;0.7]	0.84	0.99	−0.96	[−5.5;3.6]	0.54	0.75
	TTw	0.09	[−0.7;0.8]	0.80	0.99	0.15	[−0.5;0.8]	0.76	0.97
	TTf	−0.53	[−4.2;3.2]	0.38	0.61	0.15	[−0.6;0.9]	0.92	0.97
S3		0.18	[−0.9;1.2]	0.84	0.97	0.33	[−1.4;2]	0.68	0.82

Measures of repeatability of 4D flow MRI aoPWV estimates are provided: Bland-Altman mean biases and limits of agreement (LA) as well as intra class correlation coefficients (ICC). *p* values correspond to Wilcoxon rank-sum test of comparisons between aoPWV values obtained by the two observers and for the two reconstructions. S1, S2 and S3: Strategies 1, 2 and 3. TTc, TTw and TTf: transit times estimated using cross-correlation, wavelets and Fourier transforms, respectively

Analysis of the effect of reconstructed temporal resolution is summarized in the right part of Table 4, revealing that approaches using computation only in time domain (S1-TTc, S2-TTc, and S3) were less robust to the 20-phase reconstruction (ICC < 0.82) than frequency (S1-TTf, S2-TTf) or time-frequency (S1-TTw, S2-TTw) domain (ICC > 0.91) methods. Of note, at each given transit time method, the strategy with a 3D coverage performed better than the 2D plans strategy.

### Comparisons between the various PWV estimates

**Fig. 4:**
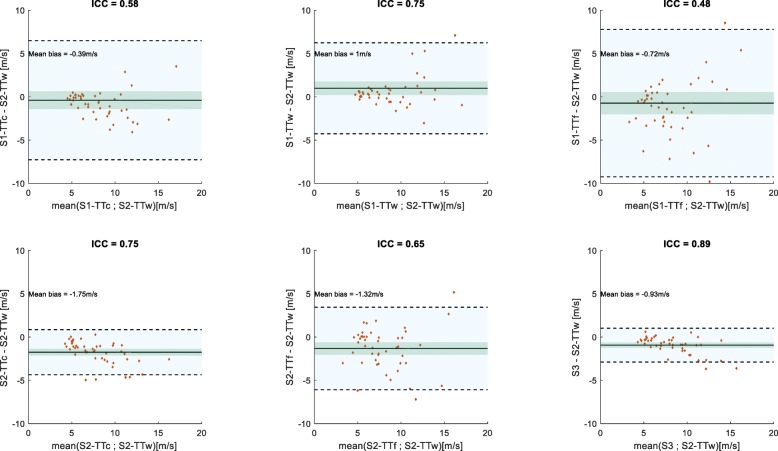
Bland-Altman analysis between S2-TTw and the remaining 4D flow CMR-derived aortic PWV. Mean bias (solid lines), limits of agreements (shaded light blue region and dotted lines) and confidence intervals (shaded green region) are showed. Intra-class correlation coefficients (ICC) are provided above each plot. S1, S2 and S3: Strategies 1, 2 and 3, respectively. TTc, TTw and TTf: transit times estimated using cross-correlation, wavelets and Fourier transforms, respectively

Table 5 summarizes results of the Bland-Altman analyses for comparisons of the 4D flow CMR aoPWV methods with Cf-PWV and BH-PWV. The corresponding plots are shown and commented in the Additional file 1. For comparisons against Cf-PWV, mean bias was close to zero or negative, in line with higher stiffness of peripheral arteries as compared with central elastic arteries. For comparisons against ascending aortic BH-PWV, mean bias was close to zero or positive, highlighting the highest elasticity of the most proximal aortic segment. Comparison between the two 4D flow CMR aoPWV methods (S2-TTw and S3) that were shown to be superior to other estimates, in terms of reproducibility as well as associations with Cf-PWV, BH-PWV, age, and LV mass-to-volume ratio, resulted in the highest ICC and the narrowest limits of agreement (Fig. 4).

**Table 5:** Bland-Altman analyses for comparisons of the 4D flow CMR aoPWV methods with Cf-PWV and BH-PWV

		Cf-PWV		BH-PWV	
		Mean bias (m/s)	LA (m/s)	Mean bias (m/s)	LA (m/s)
S1	TTc	−0.9	[−9.1;7.3]	0.9	[−8.3;10.1]
	TTw	0.5	[−7.3;8.3]	2.2	[−5.6;10]
	TTf	−1.2	[−10.4;8.0]	0.5	[−9.3;10.3]
S2	TTc	−2.3	[−6.4;1.8]	−0.5	[−6.8;5.8]
	TTw	−0.5	[−5.4;4.4]	1.2	[−4.9;7.3]
	TTf	−1.8	[−7.4;3.8]	−0.1	[−7.5;7.3]
S3		−1.4	[−5.3;2.5]	0.3	[−5.6;6.2]

Bland-Altman mean biases and limits of agreement (LA) for comparisons between 4D flow MRI aoPWV values and carotid-femoral PWV (Cf-PWV) as well as the ascending aorta Bramwell-Hill PWV (BH-PWV). Bias was defined as 4D flow MRI aoPWV – Cf-PWV or BH-PWV. S1, S2 and S3: Strategies 1, 2 and 3, respectively. TTc, TTw and TTf: transit times estimated using cross-correlation, wavelets and Fourier transforms, respectively

## Discussion

This study provides a comparison of the main methods available in the literature for aortic PWV estimation, using 4D flow CMR in 47 healthy subjects. The significant associations with age and the non-invasive reference applanation tonometry-derived Cf-PWV (*p* < 0.001) demonstrated the consistency of all 4D flow CMR-derived aoPWV estimates. Methods taking into account the whole 3D aortic spatial coverage, specifically the method based on the wavelet transform for transit time estimation were found to be superior to the other approaches, as revealed by 1) their stronger associations with Cf-PWV, Bramwell-Hill model-derived AAo PWV, age, and LV-mass-to-volume ratio as well as 2) their higher reproducibility and robustness to lower reconstructed temporal resolution. Interestingly, correlations of 4D flow CMR aoPWV methods, which account for the full 3D coverage, with age and LV mass-to-volume ratio were in the same range or even slightly higher than those obtained when using Cf-PWV, and remained significant after adjustment for the main confounders.

Aortic PWV values obtained in our study from 4D flow data are in a similar range using all methods. Furthermore, comparison against the widely available 2D PC-CMR aortic arch PWV values estimated in large populations in the literature revealed no substantial differences. Indeed, normal values for aortic arch PWV summarized by Kawel-Boehm et al. [43] were 3.9 ± 1.1 m/s for age range 30–39 years, 5.6 ± 1.4 m/s for 40–49 years, 7.2 ± 2.3 m/s for 50–59 years, 9.7 ± 2.9 m/s for 60–69 years, 11.1 ± 4.6 m/s for age ≥ 70 years. Normal values for 4D flow CMR-derived aoPWV are not available yet since the majority of previous studies was performed on small groups or pathological individuals while using various methods. Nevertheless, despite differences in population age, our values were in the same range or only slightly higher than those provided in a recent 4D flow study [44] while using similar TT-based approaches (*n* = 8, age = 23 ± 2 years: PWV = 5.7 ± 0.7 m/s when using Fourier analysis and PWV = 5.5 ± 0.7 m/s when using cross-correlation; *n* = 8, age = 58 ± 2 years: PWV = 9.3 ± 1.3 m/s when using Fourier analysis and PWV = 8.9 ± 1.4 m/s when using cross-correlation).

Our choice of the tested methods for aoPWV estimation in this study was based on previous 2D PC-CMR and 4D flow CMR studies. Indeed they have demonstrated that: 1) approaches based on a single point of the flow curve (foot or peak) are hampered by low velocity to noise ratio or by the effects of wave reflection [24, 36, 45], 2) frequency and time-frequency domain methods for TT estimation are more robust to low temporal resolution [37, 38], and 3) approaches taking into account volumetric flow data along the aorta are more robust than methods considering only two measurement sites [20, 24]. The high reproducibility of PWV previously shown in 2D PC-CMR studies was not found in our 2D-like strategy (S1), which resulted in sizeable Bland-Altman limits of agreements in our data. Such lower performances of the 2D-like strategies in our study might be explained by the static nature of the 4D flow CMR aortic lumen segmentation through time, contrary to an automated dynamic time-resolved segmentation in 2D PC-CMR, which would hamper the AAo velocity curves, as well as by the lower spatial resolution of 4D flow as compared to 2D PC-CMR data, which would highly affect the distal descending aorta because of its small cross-section.

In agreement with previous findings [36, 37], systolic upslope-based methods were more consistent than wave-based methods especially when using the 3D strategy, as revealed by the comparison between wavelet or cross-correlation and Fourier approaches in terms of associations with age and LV mass to volume ratio. This can be explained by the fact that the early systolic upslope is less distorted by wave reflexion than late systolic and diastolic phases of the flow curve [35, 36]. Besides, methods considering the volumetric coverage of 4D flow CMR data were more reliable than those based on two planes only.

Original features of our study include semi-automated segmentation of the aorta and an automated positioning of flow measurement planes perpendicular to the centreline. In addition, comparisons against the CMR-independent tonometry Cf-PWV and 4D flow CMR-independent ascending aortic BH-PWV measures, as well as the assessment of physiological associations with LV mass-to-volume ratio and age were reported. Our aoPWV measurements confirmed physiological knowledge on arterial stiffening gradient from the central aorta to peripheral arteries, resulting in the lowest values for the ascending aortic Bramwell-Hill method, intermediate values for the 4D flow CMR methods over the whole aorta, and the highest values for the carotid to femoral arteries PWV. This stiffness gradient phenomenon was more marked in younger subjects with highly elastic central arteries than in elderly subjects, because of stiffness homogenisation from central arteries towards the periphery with ageing.

Furthermore, comparison to Cf-PWV and BH-PWV revealed the superiority of the plane fitting method as well as wavelet TT-based approach previously shown using 2D-PC CMR to be more robust to low temporal resolution [37]. Our findings further confirm such robustness of the wavelet-based method to 20- vs. 50-reconstructed time frames. In addition, such method was strongly associated with age even in subjects ≥50 years, in whom we expect increased aortic stiffness and thus decreased TT when compared to younger subjects. Although the wavelet-based approach resulted in the highest performances when compared to the plane fitting approach, its implementation requires tuning of parameters such as the mother wavelet and sampling frequency. However, one might highlight that the same parameters than those previously described using 2D-PC CMR data [37] were used in each subject for the wavelet method in the present study.

Reliability of our 4D flow CMR aoPWV measurement was also demonstrated by the positive association with LV mass-to-volume ratio. Indeed, it is known that in the process of healthy aging, arterial stiffening is associated with increased LV afterload and subsequent LV hypertrophic remodeling, which was measured in our study through the LV mass to end-diastolic volume ratio [30]. Such association was independent of age, systolic blood pressure, BMI, and gender, revealing that 4D flow CMR aoPWV is an independent marker of LV alteration in the process of healthy aging. Gender was also an independent correlate of LV mass-to-volume ratio, in agreement with a previous study [46]. Accordingly, evaluation of aortic stiffness using 4D flow CMR can provide mechanistic knowledge to improve understanding of ventricular-arterial coupling.

The main limitation of our study is the lack of a direct aortic PWV gold standard measurement as provided by catheterization [10]. However such invasive procedure was not feasible in healthy subjects. Moreover, associations with the non-invasive reference Cf-PWV [1, 7, 29], and with physiological criteria such as age and LV mass-to-volume ratio were used to compare the performances of 4D flow CMR aoPWV methods. Since 4D flow CMR is limited by low spatial resolution and signal to noise ratio for low velocities, time-resolved segmentation was not available. Accordingly, a fixed segmentation on an enhanced aortic angiogram, reconstructed from peak systolic phases, was used along with mean velocity curves rather than flow curves. In the future, a time-resolved segmentation would be necessary for an accurate estimation of aortic flow along the cardiac cycle. Such segmentation would require an evolvement of 4D flow CMR sequences with the use of higher spatial resolution and multiple encoding velocities to improve velocity to noise ratio especially in regions with low velocities or the use of motion-compensated compressed-sensing techniques which were shown to improve the quality of 4D flow images [47, 48]. To enhance contrast and signal to noise ratio, contrast agent is often used in 4D flow CMR. However, since standard CMR exams in clinical routine usually include LGE and/or post-contrast T1, 4D flow CMR acquisition can be interleaved while waiting for such tissue characterization sequences to be acquired. Another limitation is the lack of test-retest variability assessment of the 4D flow CMR aoPWV due to non-available data. However, one might highlight that the main goal of our study was to isolate the technical reliability of 4D flow CMR aoPWV from the robustness of 4D flow CMR to hemodynamic changes over time. Thus, the proposed 4D flow CMR techniques were rather compared in terms of inter-observer reproducibility and associations with 4D flow CMR-independent aoPWV measures such as the tonometric Cf-PWV and the cine bSSFP-derived BH-PWV as well as physiological association with age and LV mass-to-volume ratio.

## Conclusion

Aortic PWV estimation in healthy subjects from 4D flow CMR data while considering the entire volumetric coverage of the aorta and a time-frequency wavelet approach for transit time calculation was reproducible, robust to lower reconstructed temporal resolution and reliable, as revealed by the strong associations with the well-established reference Cf-PWV, BH-PWV and physiological criteria such as age and LV mass-to-volume ratio. The LV and the aorta being simultaneously acquired in the same hemodynamic conditions when using 4D flow CMR, the derived aortic PWV might help for a better understanding of LV – aortic coupling.

## Supplementary information

**Additional file 1.** Bland-Altman plots for comparisons of 4D flow MRI aoPWV with Cf-PWV and BH-PWV.

## Data Availability

The datasets used and/or analysed during the current study are available from the corresponding author on reasonable request.

## Abbreviations

AAo: Ascending aorta
aoPWV: Aortic pulse wave velocity
BH: Bramwell-Hill
BMI: Body mass index
bSSFP: Balanced steady-state free precession
Cf: Carotid-femoral
CMR: Cardiovascular magnetic resonance imaging
D: Distance
DAo: Descending aorta
DBP: Diastolic blood pressure
ECG: Electrocardiogram
EDV: End-diastolic volume
ESV: End-systolic volume
ICC: Intra-class correlation coefficient
LV: Left ventricle/left ventricular
LVM: Left ventricular mass
PC: Phase-contrast
PP: Pulse pressure
PWV: Pulse wave velocity
SBP: Systolic blood pressure
TT: Transit time
