# Supplementary material for: Comparison of different methods for the estimation of aortic pulse wave velocity from 4D flow cardiovascular magnetic resonance
Source: J Cardiovasc Magn Reson. 2019 Dec 12;21:75. doi: 10.1186/s12968-019-0584-x (PMC6907267; doi:10.1186/s12968-019-0584-x)
Supplement: Supplementary file 1 — Additional file 1. Bland-Altman plots for comparisons of 4D flow MRI aoPWV with Cf-PWV and BH-PWV. [file 12968_2019_584_MOESM1_ESM.docx]

Bland-Altman plots for comparisons of the 4D flow MRI aoPWV methods with Cf-PWV and BH-PWV are provided in the figure below.

For comparisons against Cf-PWV, mean bias was close to zero or negative, in line with higher stiffness of peripheral arteries as compared with central elastic arteries. This phenomenon is more pronounced in young subjects in whom the a high elastic gradient from central arteries towards periphery is expected, while Cf-PWV appears to be equal to or even smaller than 4D flow MRI aoPWV in the elderly subject. This latter result can be explained by a generalized loss in arterial elastic properties along the arterial tree with age but also by a possible underestimation of Cf-PWV in the elderly subjects as a consequence of distance measurements errors as its assessment over the body surface neglects arterial tortuosity.

For comparisons against ascending aortic BH-PWV, mean bias was close to zero or positive and the majority of the subjects are above the zero line especially for S2-TTw and S3, highlighting the highest elasticity of the most proximal aortic segment.

**Additional file figure legend. Bland-Altman plots for comparisons of 4D flow MRI aoPWV with Cf-PWV and BH-PWV.**

Mean bias (solid lines), limits of agreements (shaded light blue region and dotted lines) and confidence intervals (shaded green region) are showed. Intra-class correlation coefficients (ICC) are provided above each plot. S1, S2 and S3: Strategies 1, 2 and 3, respectively. TTc, TTw and TTf: transit times estimated using cross-correlation, wavelets and Fourier transforms, respectively. Cf-PWV: carotid-femoral PWV, BH-PWV: Bramwell-Hill PWV.
